# Highlight report: Toxicogenomics atlas of rat hepatotoxicants

**DOI:** 10.17179/excli2018-2000

**Published:** 2018-12-20

**Authors:** Florian Seidel

**Affiliations:** 1 Leibniz Research Centre for Working Environment and Human Factors (IfADo), Ardeystr. 67, 44139 Dortmund

## ⁯

Transcriptomics has developed into an invaluable tool in chemical hazard identification (Godoy et al., 2013[5], 2015[7], 2018[6]; Lohr et al., 2015[13]; Ellinger-Ziegelbauer et al., 2011[3]; Waldmann et al., 2014[20]; Balmer et al., 2014[1]). The pattern of deregulated genes in exposed cells gives first evidence of the involved mechanisms of toxicity (Rempel et al., 2015[15]; Stemmer et al., 2007[17]; Leist et al., 2017[12]; Rodrigues et al., 2018[16]). A milestone in this field of research was the establishment of the toxicogenomics directory of chemically exposed human hepatocytes (Grinberg et al., 2014[10]). A key message of this study was that stereotypical expression responses exist, whereby a similar set of genes is deregulated after exposure of human hepatocytes to different compounds. A relatively large fraction of these stereotypical stress response genes are also up- or downregulated in human liver disease, such as non-alcoholic steatohepatitis, cirrhosis or hepatocellular cancer (Grinberg et al., 2014[10]). While the human toxicogenomics directory has been widely used for follow-up studies, a similar database for rat hepatocytes has not yet been established. 

To bridge this gap, Marianna Grinberg and colleagues from Dortmund University recently published the corresponding directory of rat hepatotoxicants (Grinberg et al., 2018[9]). Laboratory animals offer the advantage that liver tissue after exposure to test compounds can be compared to cultivated hepatocytes exposed to the same compounds. For this purpose, the authors analyzed microarray expression data from 162 test substances that were tested in a concentration-dependent manner in rat livers *in vivo* and in cultivated hepatocytes. Based on this comprehensive data set genes were analyzed that showed a similar response *in vitro *and* in vivo*. Next, genes were identified that were most frequently deregulated by the test compounds. This resulted in seven genes with the highest coverage of compounds (Cyp1a1, Vgt2b1, Cdkn1a, Mdm2, Aldh1a1, Cyp4a3 and Ehhadh). Analysis of these genes in hepatocytes incubated with compounds not present in the above mentioned set of 162 test substances showed that at least one of these seven genes was also deregulated in the set of independent compounds.

Currently, hepatotoxicity represents a major research field in toxicology (Vartak et al., 2016[19]; Godoy et al., 2016[8]; Bolt, 2017[2]; Hassan, 2016[11]). Techniques for the reliable identification of compounds that will induce liver injury of humans are urgently needed (Stöber, 2016[18]; Ghallab, 2017[4]; Paech et al., 2017[14]). In this field of research the recently established human and rat toxicogenomics directories represent invaluable resources. 
